# Differences in reproductive toxicology between alopecia drugs: an analysis on adverse events among female and male cases

**DOI:** 10.18632/oncotarget.12617

**Published:** 2016-10-12

**Authors:** Min Wu, Qingxiong Yu, Qingfeng Li

**Affiliations:** ^1^ Department of Plastic and Reconstruction Surgery, Shanghai Ninth People's Hospital, Shanghai Jiao Tong University School of Medicine, Shanghai, China

**Keywords:** reproductive toxicity, alopecia, finasteride, minoxidil, FDA adverse event reporting system, Pathology Section

## Abstract

Alopecia is a dermatological condition with limited therapeutic options. Only two drugs, finasteride and minoxidil, are approved by FDA for alopecia treatment. However, little is known about the differences in adverse effects between these two drugs. We examined the clinical reports submitted to the FDA Adverse Event Reporting System (FAERS) from 2004 to 2014. For both female and males, finasteride was found to be more associated with reproductive toxicity as compared to minoxidil. Among male alopecia cases, finasteride was significantly more concurrent with several forms of sexual dysfunction. Among female alopecia cases, finasteride was significantly more concurrent with harm to fetus and disorder of uterus. In addition, drug-gene network analysis indicated that finasteride could profoundly disturb pathways related to sex hormone signaling and oocyte maturation. These findings could provide clues for subsequent toxicological research. Taken together, this analysis suggested that finasteride could be more liable to various reproductive adverse effects. Some of these adverse effects have yet to be warned in FDA-approved drug label. This information can help improve the treatment regimen of alopecia and post-marketing regulation of drug products.

## INTRODUCTION

Alopecia refers to the unwanted loss of hair from the head or body, which affects both men and women [1]. Rather than physiological injury, the predominant impact of alopecia is psychological stress caused by the change in appearance. Psychological problems in some cases can deteriorate till the onset of severe symptoms [2]. Therefore, many affected subjects have strong willing to arrest progression of hair loss and stimulate hair growth, thus motivating basic research and drug development related to alopecia. The etiologies of alopecia are still largely unknown, but a series of hypotheses has been proposed to explain the causes [3]. Alopecia areata is considered an autoimmune disease, in which the immune system mistakenly attacks hair follicles [4]. Androgenic alopecia (a.k.a. male pattern baldness) may be a result of hair follicles miniaturization [5, 6] and microinflammation [7].

As a result of complicated etiologies, there are very limited therapeutic options so far. Finasteride and minoxidil are the only two drugs approved by Food and Drug Administration (FDA) for alopecia treatment. Finasteride oral tablet is approved for the treatment of benign prostatic hyperplasia and male pattern baldness. As a 5α-reductase inhibitor, finasteride can prevent the conversion of testosterone to dihydrotestosterone, thus reducing androgen activity in the scalp [8]. Minoxidil is originally designed as an antihypertensive drug. Ever since hair regrowth is found as a side effect of treatment for hypertension, minoxidil has been broadly used for the topical treatment of alopecia. Many mechanisms of action have been proposed to explain the regrowth effect of minoxidil, including stimulation of blood flow in scalps [9], development of dermal papilla vascularization [10], enhanced hair follicular cycles [11] and potassium channel conductance [12]. Finasteride and minoxidil are effective in only a proportion of alopecia cases. But long after their discovery, there are still no new FDA-approved remedies.

Since finasteride and minoxidil are the two major treatments for alopecia, it is naturally a compelling issue to understand their clinical differences in both efficacy and risks. In addition, it is essential for regulators, clinicians and consumers to understand the drug effects in men and women, respectively [13, 14]. The differences in therapeutic effect between these two drugs were found to be sex-dependent. For male cases, a series of comparative studies suggested a better efficacy of finasteride [15–17]. In contrast, for female cases, several clinical studies suggested that finasteride was generally ineffective [18–20], while the efficacy of minoxidil was repeatedly reported [21, 22]. On the other hand, however, the toxicological differences between finasteride and minoxidil were rarely addressed in previous studies, which was partially due to the difficulty in collecting clinical data about adverse effects from alopecia cases [23].

The FDA Adverse Event Reporting System (FAERS) is a computerized information database established by U.S. Government, which restores reports of adverse events spontaneously submitted by patients and healthcare professionals. Ever since 2004, the continuous operation of FAERS has achieved an enormous data collection, so as to support FDA's post-marketing safety surveillance for approved drug products. Once a pharmacovigilance signal was detected, a more rigorous investigation could be conducted. In the meantime, FAERS also promoted clinical studies worldwide [24] and lead to a series of publications regarding various drugs and adverse effects [25–27]. Because of the increasingly broad application of FAERS data, the openFDA initiative was officially launched in June 2014, which provided an official application programming interface (API) to access the raw data of adverse event reports in a structured format.

In the present study, we were enabled by openFDA to retrieve adverse events reported to FAERS by alopecia cases. By comparing the adverse events of finasteride and minoxidil, we identified a series of common adverse reactions that were significantly more concurrent with one drug than the other. In particular, many of these adverse reactions were directly related to female or male reproductive system. The differences in reproductive toxicology between alopecia drugs reiterated the precautions of clinical drug use and warranted subsequent research on the underlying toxicological mechanisms.

## RESULTS

### The gender composition of case reports

We primarily examined the gender composition of alopecia cases. The numbers of reports for both female and male cases were counted (Table 1), while the reports without gender information were excluded for analysis (see Methods). Adverse events of male patients accounted for only 38.3% of the total FAERS reports. But among alopecia cases, the reports of male cases comprised a significantly higher proportion. Nearly half of minoxidil related reports (i.e., 48.2%) and most finasteride related reports (i.e., 97.2%) were submitted by male cases. Such an highly biased gender composition of finasteride related reports should be attributed to the fact that finasteride was approved for alopecia in men only [28].

**Table 1:** The number of reports for female and male alopecia patients

	Male	Female	Percentage of Male	ORM-F^1^	*P*-value^2^
All FAERS Reports	1750808	2818346	38.3%	-	-
Finasteride for alopecia treatment	2076	60	97.2%	55.70	0
Minoxidil for alopecia treatment	92	99	48.2%	1.50	0.0058

1 ORM-F > 1.0 indicates that the proportion of male cases is higher than the overall level of all FAERS reports.

2 *P*-value, suggesting the significance of difference in gender composition, is determined using the Fisher's exact test.

Then, we scrutinized the top 10 most commonly reported adverse events among female and male alopecia cases exposed to either finasteride or minoxidil (Table 2). It appeared that female and male cases were prone to different adverse effects. In addition, a number of adverse effects were directly related to reproductive system, such as ‘abortion induced’ among females and ‘erectile dysfunction’ among males. Then, we compared the reports of finasteride and minoxidil, so as to find whether certain adverse effects were significantly more associated with one drug than the other. Due to the intrinsic gender difference in physiology, we analyzed the data of female and male cases separately.

**Table 2:** The top 10 most commonly reported adverse events among female and male alopecia patients using finasteride or minoxidil

Rank	Finasteride		Minoxidil	
	Female	Male	Female	Male
1	Abortion Induced	Erectile Dysfunction	Swelling Face	Erectile Dysfunction
2	Abortion Spontaneous	Sexual Dysfunction	Dermatitis Contact	Depression
3	Paternal Drugs Affecting Fetus	Depression	Arthralgia	Dizziness
4	Uterine Cervix Stenosis	Anxiety	Palpitations	Anxiety
5	Menstruation Irregular	Cognitive Disorder	Dizziness	Libido Decreased
6	Menorrhagia	Libido Decreased	Nausea	Hypoesthesia
7	Endometrial Hypertrophy	Loss Of Libido	Tachycardia	Headache
8	Phalangeal Agenesis	Fatigue	Weight Increased	Dermatitis Contact
9	Fatigue	Semen Volume Decreased	Visual Acuity Reduced	Chorioretinopathy
10	Arthritis	Ejaculation Disorder	Pruritus	Skin Disorder

### The adverse events among male cases

Estimating safety risk from spontaneous reports is not straightforward, since the number of reported events is correlated to the total number of patients taking the drug. For example, if a blockbuster drug is used by a large number of patients, the number of adverse events of that drug may naturally be relatively large. For that reason, we calculated the proportion of a certain adverse effect in the total reports of a drug, in order to normalize the difference in the amount of usage between individual drugs. Thus, the proportion value of finasteride was compared to that of minoxidil, which lead to the proportional reporting ratio (PRR) [29], along with 95% confidence interval (CI) and significance level (see Methods). As a qualitative indicator, PRR showed which drug was more concurrent with a certain adverse effect.

Among male cases, finasteride was found to be more frequently reported for reproductive adverse effects (Table 3). For instance, ‘erectile dysfunction’ was reported by 50.39% of the cases receiving finasteride treatment, while this proportion for minoxidil was only 4.35%. A PRR of 11.59 (95 CI: 4.44 - 30.25) indicated a significant difference between these two drugs (*P* = 8.31×10^−12^). Likewise, ‘ejaculation disorder’ (PRR = 7.67; *P* = 0.013), ‘libido decreased’ (PRR = 21.98; *P* = 1.02×10^−7^), ‘loss of libido’ (PRR = 13.52; *P* = 8.22×10^−5^), ‘semen volume decreased’ (PRR = 8.51; *P* = 5.78×10^−3^) and ‘sexual dysfunction’ (not reported for minoxidil; *P* = 2.54×10^−15^) were also more concurrent with finasteride as compared to minoxidil. These results suggested that finasteride should be used by males with more caution.

**Table 3:** The comparison between adverse events reported by male alopecia patients exposed to finasteride and minoxidil

Adverse Effect	Drug	Affected Cases	Total Cases	Proportion^1^	PRR (95% CI)^2^	*P*-value^3^
Anxiety	Finasteride	671	2076	32.32%	29.74 (4.23 - 209.10)	4.17×10^−10^***
	Minoxidil	1	92	1.09%		
Chorioretinopathy	Finasteride	3	2076	0.14%	0.02 (0.01 - 0.08)	5.39×10^−7^***
	Minoxidil	6	92	6.52%		
Cognitive Disorder	Finasteride	607	2076	29.24%	N/A	1.75×10^−10^***
	Minoxidil	0	92	0.00%		
Depression	Finasteride	727	2076	35.02%	10.74 (3.52 - 32.74)	5.55×10^−9^***
	Minoxidil	3	92	3.26%		
Dermatitis Contact	Finasteride	8	2076	0.39%	0.06 (0.02 - 0.17)	1.58×10^−5^***
	Minoxidil	6	92	6.52%		
Dizziness	Finasteride	67	2076	3.23%	0.59 (0.24 - 1.43)	0.24
	Minoxidil	5	92	5.43%		
Ejaculation Disorder	Finasteride	173	2076	8.33%	7.67 (1.09 - 54.15)	0.013*
	Minoxidil	1	92	1.09%		
Erectile Dysfunction	Finasteride	1046	2076	50.39%	11.59 (4.44 - 30.25)	8.31×10^−12^***
	Minoxidil	4	92	4.35%		
Fatigue	Finasteride	232	2076	11.18%	5.14 (1.30 - 20.35)	6.88×10^−3^**
	Minoxidil	2	92	2.17%		
Headache	Finasteride	84	2076	4.05%	0.62 (0.28 - 1.38)	0.28
	Minoxidil	6	92	6.52%		
Hypoesthesia	Finasteride	40	2076	1.93%	0.44 (0.16 - 1.20)	0.12
	Minoxidil	4	92	4.35%		
Libido Decreased	Finasteride	496	2076	23.89%	21.98 (3.12 - 154.62)	1.02×10^−7^***
	Minoxidil	1	92	1.09%		
Loss of Libido	Finasteride	305	2076	14.69%	13.52 (1.92 - 95.23)	8.22×10^−5^***
	Minoxidil	1	92	1.09%		
Semen Volume Decreased	Finasteride	192	2076	9.25%	8.51 (1.21 - 60.05)	5.78×10^−3^**
	Minoxidil	1	92	1.09%		
Sexual Dysfunction	Finasteride	956	2076	46.05%	N/A	2.54×10^−15^***
	Minoxidil	0	92	0.00%		
Skin Disorder	Finasteride	25	2076	1.20%	0.37 (0.11 - 1.20)	0.12
	Minoxidil	3	92	3.26%		

1 The proportion value is computed as the number of affected cases divided by the number of total cases.

2 A PRR significantly greater (or lower) than 1.0 means the risk is higher for finasteride (or minoxidil).

3 *, *p* < 0.05; **, *p* < 0.01; ***, *p* < 0.001

In addition to reproductive toxicity, some psychiatric reactions were not emphasized in FDA-approved drug label [28] but frequently reported by cases exposed to finasteride, including ‘anxiety’ (PRR = 29.74; *P* = 4.17×10^−10^), ‘depression’ (PRR = 10.74; *P* = 5.55×10^−9^) and ‘cognitive disorder’ (not reported for minoxidil; *P* = 1.75×10^−10^). Only a few adverse effects were apparently more reported by minoxidil users, such as ‘dermatitis contact’ (PRR = 0.06; *P* = 1.58×10^−5^) and ‘chorioretinopathy’ (PRR = 0.02; *P* = 5.39×10^−7^). These adverse effects should also be taken into consideration in selecting the appropriate treatment.

### The adverse events among female cases

On the other hand, we also examined the adverse effects among female cases. Even though finasteride has not been officially approved for use by women, a number of female cases exposed to finasteride were still found in FAERS data. Compared with minoxidil, finasteride exhibited significantly negative effect on female reproductive system (Table 4). Fetal toxicity, characterized by ‘abortion induced’ (PRR = 9.07; *P* = 1.93×10^−3^), ‘abortion spontaneous’ (PRR = 6.60; *P* = 0.016) and ‘paternal drugs affecting fetus’ (not reported for minoxidil; *P* = 8.89×10^−3^), was more concurrent with finasteride than minoxidil. Moreover, finasteride may affect uterus, leading to more reports of ‘uterine cervix stenosis’ (not reported for minoxidil; *P* = 0.022) and ‘endometrial hypertrophy’ (not reported for minoxidil; *P* = 0.022). These results revealed the safety risks of unapproved use of finasteride in female cases, especially in pregnant women. In contrast, minoxidil-biased risk was not widely detected. ‘Swelling face’, as a relatively minor safety concern, was the only adverse effect significantly more reported by female cases exposed to minoxidil (not reported for finasteride; *P* = 0.027).

**Table 4:** The comparison between adverse events reported by female alopecia patients exposed to finasteride and minoxidil

Adverse Effect	Drug	Affected Cases	Total Cases	Proportion^1^	PRR (95% CI)^2^	*P*-value^3^
Abortion Induced	Finasteride	11	60	18.33%	9.07 (2.08 - 39.53)	1.93×10^−3^**
	Minoxidil	2	99	2.02%		
Abortion Spontaneous	Finasteride	8	60	13.33%	6.60 (1.45 - 30.06)	0.016*
	Minoxidil	2	99	2.02%		
Arthralgia	Finasteride	3	60	5.00%	0.55 (0.15 - 1.95)	0.54
	Minoxidil	9	99	9.09%		
Arthritis	Finasteride	3	60	5.00%	N/A	0.057
	Minoxidil	0	99	0.00%		
Dermatitis Contact	Finasteride	2	60	3.33%	0.37 (0.08 - 1.66)	0.33
	Minoxidil	9	99	9.09%		
Dizziness	Finasteride	2	60	3.33%	0.41 (0.09 - 1.87)	0.33
	Minoxidil	8	99	8.08%		
Endometrial Hypertrophy	Finasteride	4	60	6.67%	N/A	0.022*
	Minoxidil	0	99	0.00%		
Fatigue	Finasteride	3	60	5.00%	0.99 (0.25 - 3.99)	1.00
	Minoxidil	5	99	5.05%		
Menorrhagia	Finasteride	4	60	6.67%	6.60 (0.76 - 57.68)	0.077
	Minoxidil	1	99	1.01%		
Menstruation Irregular	Finasteride	4	60	6.67%	6.60 (0.76 - 57.68)	0.077
	Minoxidil	1	99	1.01%		
Nausea	Finasteride	2	60	3.33%	0.47 (0.10 - 2.19)	0.49
	Minoxidil	7	99	7.07%		
Palpitations	Finasteride	0	60	0.00%	0.00	0.52
	Minoxidil	8	99	8.08%		
Paternal Drugs Affecting Fetus	Finasteride	5	60	8.33%	N/A	8.89×10^−3^**
	Minoxidil	0	99	0.00%		
Phalangeal Agenesis	Finasteride	3	60	5.00%	N/A	0.057
	Minoxidil	0	99	0.00%		
Pruritus	Finasteride	1	60	1.67%	0.33 (0.04 - 2.76)	0.41
	Minoxidil	5	99	5.05%		
Swelling Face	Finasteride	0	60	0.00%	0.00	0.027*
	Minoxidil	9	99	9.09%		
Tachycardia	Finasteride	0	60	0.00%	0.00	0.087
	Minoxidil	6	99	6.06%		
Uterine Cervix Stenosis	Finasteride	4	60	6.67%	N/A	0.022*
	Minoxidil	0	99	0.00%		
Visual Acuity Reduced	Finasteride	0	60	0.00%	0.00	0.16
	Minoxidil	5	99	5.05%		
Weight Increased	Finasteride	0	60	0.00%	0.00	0.16
	Minoxidil	5	99	5.05%		

1 The proportion value is computed as the number of affected cases divided by the number of total cases.

2 A PRR significantly greater (or lower) than 1.0 means the risk is higher for finasteride (or minoxidil).

3 *, *p* < 0.05; **, *p* < 0.01

### Drug-gene network and functional enrichment analysis

**Figure 1:**
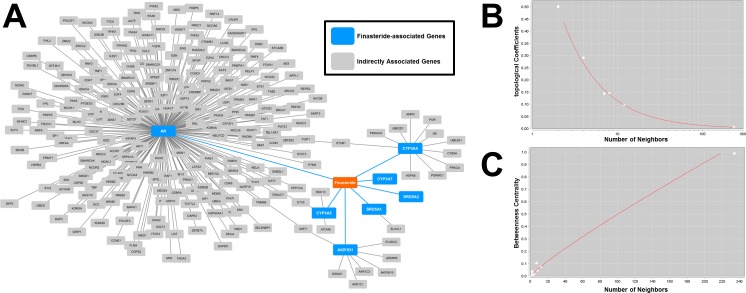
Visualization and analysis of drug-gene network **A.** The finasteride-associated genes and indirectly associated genes were integrated into a network. A node denoted a drug (i.e., finasteride) or a gene. An edge represented an interaction between two nodes. **B.** The topological coefficients followed the power law distributions, with an R^2^ = 0.99. **C.** The betweenness centrality followed the power law distributions, with an R^2^ = 0.84.

The aim of this study was not only to identify the relative higher risk of finasteride, but more importantly, to better understand how finasteride interfered with the normal function of reproductive system. Almost all drugs act through the interactions with numerous proteins in human body encoded by different genes. In recent years, the integrated analysis of drug-gene interactions has provided many applications in toxicological research [30]. To extend our knowledge of reproductive toxicity, we constructed a drug-gene network [31] and explored a number of genes directly or indirectly interrupted by finasteride (see Materials and Methods). First, we searched the PharmGKB database (https://www.pharmgkb.org/) and the DrugBank database (http://www.drugbank.ca/) to extract the finasteride-associated genes (FAGs) with known pharmacokinetic and pharmacodynamic evidences. Then, the FAGs were overlaid into the context of human protein-protein interactions (PPIs) to recruit their neighbor proteins, whose encoding genes were defiend as indirectly associated genes (IAGs) of finasteride. At last, all FAGs and IAGs were integrated into a network with 260 nodes (259 genes and 1 drug) and 262 edges, which characterized the toxicology of finasteride (Figure 1A). It is common knowledge that the topology of true biological networks is obviously different from that of random networks. The topological coefficients (Figure 1B) and betweenness centrality (Figure 1C) of the finasteride-centered network approximately followed power law distributions [32]. This suggested that a few hubs in this network linked to most of other nodes, which is one of the most distinguishing characteristics of scale-free biological networks [33].

To interpret the biological importance of this network, we queried the FAGs and IAGs in the WebGestalt online server (see Materials and Methods). Enrichment analyses for the KEGG pathways and Gene Ontology (GO) terms were performed (Table 5). Regarding reproductive toxicity in men, we found that the FAGs and IAGs were significantly enriched in the ‘steroid hormone biosynthesis’ pathway (*P* = 7.16×10^−9^), ‘intracellular steroid hormone receptor signaling pathway’ (*P* = 1.24×10^−47^) and ‘androgen receptor signaling pathway’ (*P* = 2.00×10^−44^). These findings make very important sense, since steroid hormones, specially androgen, are well known to play an fundamental role in maintaining male sexual function [34, 35]. Regarding reproductive toxicity in women, we identified two KEGG pathways that were highly enriched with FAGs and IAGs, including ‘oocyte meiosis’ (*P* = 1.43×10^−5^) and ‘progesterone-mediated oocyte maturation’ (*P* = 3.27×10^−5^). Inadequate secretion of progesterone has been proposed as a cause of spontaneous abortion [36, 37]. These results indicated that finasteride could profoundly affect the normal reproductive function by acting on specific key genes in relevant pathways.

**Table 5:** The functional annotations enriched with genes interrupted by finasteride

	Genes in the category	OR of enrichment	Adjusted *P*-value^1^
**GO Terms**			
Intracellular steroid hormone receptor signaling pathway (GO:0030518)	EP300, PHB, SMARCA4, KAT5, UBE2I, SRC, CCNE1, PARK7, TRIM68, TADA3, CALR, PIAS1, NR0B1, RB1, UBE3A, NCOA6, CTNNB1, RNF4, GRIP1, MED24, RAN, MED1, FKBP4, NCOA2, SIRT1, SKP2, FHL2, BRCA1, CDK7, KDM3A, NCOA3, RNF6, NCOA4, DAXX, PMEPA1, PIAS2, HDAC1, AR, NCOA1, NR0B2, RNF14, FOXA1, TGFB1I1	24.73	1.24×10^−47^
Androgen receptor signaling pathway (GO:0030521)	EP300, PHB, SMARCA4, KAT5, CCNE1, PARK7, TRIM68, PIAS1, RB1, UBE3A, CTNNB1, RNF4, MED24, GRIP1, RAN, MED1, FKBP4, NCOA2, SIRT1, FHL2, BRCA1, CDK7, KDM3A, NCOA3, RNF6, NCOA4, PMEPA1, DAXX, PIAS2, HDAC1, AR, NR0B2, NCOA1, RNF14, TGFB1I1	32.91	2.00×10^−44^
**KEGG Pathways**			
Steroid hormone biosynthesis (hsa00140)	AKR1D1, CYP3A7, CYP3A5, AKR1C1, SRD5A2, AKR1C3, SRD5A1, CYP3A4	23.88	7.16×10^−9^
Oocyte meiosis (hsa04114)	AR, YWHAQ, PPP1CA, MAPK1, PRKACA, CCNE1, CALM1	10.45	1.43×10^−5^
Progesterone-mediated oocyte maturation (hsa04914)	HSP90AA1, CDC25B, AKT1, MAPK1, PRKACA, RAF1	11.66	3.27×10^−5^

1 The *p*-values were calculated by hypergeometric test and adjusted with the Benjamini-Hochberg procedure.

## DISCUSSION

Most alopecia cases with the intent of receiving treatment may choose either finasteride or minoxidil, since they are the two major drug products approved by FDA for marketing. Therefore, the differences between these two drugs are always a compelling clinical issue. In previous studies, a lot of efforts have been made to unveil their difference in efficacy. However, not only efficacy but also toxicity of drugs should be fully understood to determine the remedy with optimal benefit-to-risk ratio [38]. Clinical trials with only a limited number of patients and a relatively short period of treatment may not give the full picture of safety risks, which need to be supplemented by large-scale post-marketing data collected from general population. As an excellent example of post-marketing data source, OpenFDA platform provided formatted and annotated information about drug generic names, patient characteristics and adverse reactions. Such information enabled us to efficiently explore the adverse events related to finasteride and minoxidil. For both genders, finasteride was concurrent with reproductive adverse effects more often than minoxidil, which reiterated the importance of studying undesirable drug effects on reproductive system [39]. And the toxicological differences between finasteride and minoxidil should be seriously considered when selecting treatment regimen.

Among male cases, a relatively higher risk of sexual dysfunction was observed with exposure to finasteride, which corroborated the information in FDA-approved drug label [28] and the results of previous controlled trials [40]. These andrological adverse effects may be closely related to the pharmacological mechanisms of finasteride. As a drug originally developed for benign prostatic hyperplasia, finasteride could influence androgen activity, which may profoundly affect sexual function of men. Apart from reproductive toxicity in men, we also found that finasteride was more likely to induce psychiatric reactions, and minoxidil was more likely to induce dermatitis and chorioretinopathy. These results were consistent with previous clinical observation [41–43] and warranted in-depth toxicologically research.

In the meantime, reproductive toxicity of finasteride was also reported by female alopecia cases. In the FDA-approved drug label, it has been warned that finasteride may cause birth defects in fetus [28]. Our analysis showed that besides birth defects, drug-induced abortion and disorder of uterus were also significantly associated with finasteride. Although finasteride was approved for alopecia in men only, the female cases in FAERS revealed that finasteride may also be used by women under some circumstances. Usually, the negative cosmetic effect of hair loss may cause greater psychological stress in women than in men. As alopecia is becoming a clinical problem increasingly common in women [44], some female alopecia cases may have strong willing try every possible remedy, even the effect of finasteride on women remains largely unclear. This observation reminded us that more attention should be paid to unapproved treatment of alopecia. And subsequent research will be required to elucidate how finasteride may affect female reproductive system.

Accumulated researches have demonstrated that an integrative analysis of drug-gene network can provide deep insights into the molecular toxicological mechanisms of drugs. Therefore, we extracted and linked a set of human genes directly or indirectly interrupted by finasteride. The topology characteristics showed that the finasteride-centered network were scale-free just as true biological networks. In this network, we found that finasteride was closely related to androgen receptor (gene symbol AR) and several other genes, thus disturb a network significantly associated with a number of functional annotations (e.g., sex hormone signaling and oocyte maturation). These functional evidences, from the network aspect, can illustrate the toxic effect of finasteride on male and female reproductive systems, thus providing objectives for follow-up research.

Despite of valuable discoveries, some inherent limitations of FAERS data should be borne in mind. First, the number of a certain adverse effect may not directly measure the risk of drug in reality, since spontaneous reporting may not represent the completeness of case finding [45]. Moreover, even if reporting is complete, it is hardly possible to enumerate the underlying population of drugs users as the denominator. Therefore, the proportional reporting ratio that resembled a risk ratio in its distribution and interpretation was calculated as an alternative measure. Regarding the adverse reaction of interest, the reporting rate of finasteride users was compared to the rate of minoxidil users. In this way, the difference between these two drugs could be qualitatively detected. But the reliability of PRRs may still be jeopardized by confounding factors such as age, comorbidities, and prior treatment history. Second, it has been reported that finasteride and minoxidil were used in combination in some cases [16, 46]. Nevertheless, the number of adverse event reports concurrent with both finasteride and minoxidil was too low in FAERS data to achieve an acceptable statistical power. Therefore, we would suggest that other clinical data regarding the combined treatment of finasteride and minoxidil should be scrutinized to better address the safety issue of drug combination. Third, the close association between high drug dose and incidence of adverse drug reaction has been demonstrated by prior studies [47]. However, dosage information was not analyzed in the present study, because it was not clearly recorded in most FAERS reports. So we would expect the dosage factor in toxicity to be examined in other clinical data of alopecia cases. Fourth, the analysis on drug-gene interactions led to a finasteride-centered network, which provided new clues with regard to the reproductive toxicity of finasteride. The next step should involve carefully selecting specific candidate genes from the network for in-depth research, such as drug-induced differential gene expression [48] and mitochondrial DNA damage [49].

All above concerns reminded us that the current results are only the beginning, rather than a complete conclusion, of the toxicological research about alopecia treatments. First of all, while interpreting the P-values and PRRs judiciously, further large-scale cohort studies will be required to quantitatively confirm the incidence rate of various adverse effects of finasteride or minoxidil. Also, follow-up experimental research could be performed on the basis of our analysis, so as to better understand the reproductive toxicity of finasteride. For instance, it has been well known that drug could induce adverse reactions by unexpectedly interacting with off-target proteins and perturbing downstream signaling pathways. Therefore, the off-targets [50, 51] and genomic expression changes [52, 53] related to finasteride could be further explored. Moreover, it should be noticed that finasteride was approved by FDA for oral use, while minoxidil was approved for topical use. Previous research indicated that the route of administration could greatly influence the degree of toxicity, and oral administration was usually more risky than external use [54]. Because of that, it is worth trying to change the route of administration [55] or adjust the oral dosage [56] of finasteride, so as to reduce the toxic effects.

Taken together, our analysis on FAERS data provided a unique perspective on the toxicological differences between finasteride and minoxidil. In particular, finasteride was more reported for reproductive adverse effects among both women and men, some of which have yet to be warned in FDA-approved drug label. These findings should be considered to effectively improve alopecia treatment and post-marketing regulation of drug products. In addition, the present study will foster further clinical and experimental research on the underlying mechanisms of drug-induced reproductive toxicity.

## MATERIALS AND METHODS

### Query of FAERS data

The original reports restored in FAERS were queried from OpenFDA platform following the official tutorial (https://open.fda.gov/api/reference/). The events related to either finasteride or minoxidil, and submitted from January 2004 to June 2014 were retrieved. Since both finasteride and minoxidil have indication other than alopecia, the adverse events were included only if the drug indication was annotated with either of the following OpenFDA query terms: “ALOPECIA”, “ALOPECIA AREATA”, “ALOPECIA EFFLUVIUM”, “ALOPECIA SCARRING”, “ALOPECIA TOTALIS”, “ALOPECIA UNIVERSALIS”, “ANDROGENETIC ALOPECIA”, “DIFFUSE ALOPECIA”, which were coded using MedDRA terminology (http://www.meddra.org/). Taking gender difference in to consideration, the adverse events occurred among female and male cases were queried and analyzed separately. Those reports without clear information of patient's gender were not included in this analysis.

### Proportional reporting ratio (PRR)

The top 10 most commonly reported adverse events among finasteride users or by minoxidil users were examined. To compare the risk of finasteride and minoxidil, the proportional reporting ratio (PRR) was calculated. Consider a hypothetical situation in which adverse effect X was reported by A_1_ alopecia cases among all the A_0_ cases exposed to finasteride. On the other hand, the same adverse effect was reported by B_1_ cases among all the B_0_ cases exposed to minoxidil. Therefore, The PRR for adverse effect X should be computed as follows:

$$PRR_{X}=\frac{A_{1}/A_{0}}{B_{1}/B_{0}}$$

The null hypothesis was formulated as PRR_X_ = 1, suggesting that adverse effect X was not more frequently concurrent with one drug than the other. The P-value was calculated with two-tailed Fisher's exact test to determine whether the null hypothesis was valid. And the 95% confidence interval (CI) of PRR was calculated as

$$\begin{array}{lll} 95\%\;CI_{\text{lower}} & = & e^{\text{In}(PRR_{X})-1.96\times \sqrt{\frac{1}{A_{1}}+\frac{1}{B_{1}}-\frac{1}{A_{0}}-\frac{1}{B_{0}}}}\\ 95\%\;CI_{\text{upper}} & = & e^{\text{In}(PRR_{X})+1.96\times \sqrt{\frac{1}{A_{1}}+\frac{1}{B_{1}}-\frac{1}{A_{0}}-\frac{1}{B_{0}}}} \end{array}$$

A PRR significantly greater than its null value of 1 indicated a finasteride-biased risk of adverse effect X. Otherwise, a PRR significantly lower than 1 indicated a minoxidil-biased risk.

### Analysis of drug-gene network

A drug-gene network was generated with three types of nodes, including drug (i.e., finasteride), drug-associated gene, and neighbor proteins interacting with drug-associated genes. First, finasteride was queried in the PharmGKB database (https://www.pharmgkb.org/) [57] and the DrugBank database [58] to extract drug-associated genes according to various published evidences. Then, the proteins interacting with the above drug-associated genes were extracted from the BioGRID database (version 3.4.138,http://thebiogrid.org/) [59]. Finally, nodes interacting with each other were linked with an edge to construct a network. The visualization and topological analysis of this drug-gene network was deployed with the Cytoscape software (version 3.4.0,http://www.cytoscape.org/) [60]. All genes in this network were input into the WEB-based GEne SeT AnaLysis Toolkit (WebGestalt,http://bioinfo.vanderbilt.edu/webgestalt/) [61]. Hypergeometric test was performed to evaluate the statistical significance of enrichment for KEGG pathways (http://www.genome.jp/kegg/pathway.html) [62] and Gene Ontology terms (http://geneontology.org/) [63]. The raw P-values were adjusted for multiple testing with the Benjamini-Hochberg procedure.
